# Determination of the target population in early benefit assessments in Germany: challenges for non-small-cell lung cancer

**DOI:** 10.1007/s10198-020-01180-1

**Published:** 2020-03-31

**Authors:** C. ten Thoren, C. Balg, J. Gibbert, S. Mostardt, M. Ripoll, D. Schierbaum, S. Schiller, A. Schwalm

**Affiliations:** Ressort Versorgung und Gesundheitsökonomie/Dept. of Health Care and Health Economics, Institut für Qualität und Wirtschaftlichkeit im Gesundheitswesen (IQWiG), Im Mediapark 8, 50670 Köln, Germany

**Keywords:** Non-small-cell lung cancer, NSCLC, Early benefit assessment, New drugs, Target population, I10 (General), I18 (Government Policy-Regulation-Public Health)

## Abstract

**Objectives:**

Dossiers submitted for early benefit assessments in Germany also provide information on the precise determination of the target population (patients eligible for a drug). The situation is complex for non-small-cell lung cancer (NSCLC) due to highly specific therapeutic indications. Our aim was to compare the different methodological steps applied to determine the target population in dossiers on drugs for NSCLC.

**Methods:**

We analysed NSCLC dossiers assessed by the German Institute for Quality and Efficiency in Health Care (IQWiG) between 01.01.2011 and 31.12.2017. Methodological details regarding the determination of the target population were extracted and compared.

**Results:**

We analysed 23 NSCLC dossiers. In all dossiers, the target population was determined using the number of all patients with lung cancer as the basis for calculations. This patient population was further reduced in several successive steps by assuming proportions of patients with a specific characteristic (e.g. disease stage). The most important calculation steps were patients with NSCLC (*n* = 23 dossiers), with a specific disease stage (*n* = 23), with a specific tumour mutation (*n* = 14), with a specific tumour histology (*n* = 7), without prior treatment (*n*  = 15), with pretreatment in second or further treatment lines (*n*  = 17), and/or with specific pretreatments (*n*  = 9). The proportions of patients determined within the same calculation step varied considerably between dossiers.

**Discussion:**

The calculation methods applied and the target population sizes reported in NSCLC dossiers vary considerably. A consensus with regard to the databases and calculation methods used to determine the target population in NSCLC would be helpful to reduce variations.

## Introduction

Since 2011, a so-called early benefit assessment of each new drug has been required within 6 months after its market launch in Germany [1, 2]. For this purpose, the pharmaceutical company responsible must submit a dossier to the Federal Joint Committee (Gemeinsamer Bundesausschuss, G-BA), the main health technology assessment (HTA) decision-making body. The G-BA generally commissions the Institute for Quality and Efficiency in Health Care (Institut für Qualität und Wirtschaftlichkeit im Gesundheitswesen, IQWiG), the German HTA agency, to perform the assessment. The dossier submitted must contain all available information on the drug’s added benefit over standard care, a description of the patient groups eligible for treatment according to the new drug’s approval status (the target population), the number of these patients, as well as the treatment costs of the new drug and of standard care [2]. The results of the assessment subsequently can serve as the basis for price negotiations between the umbrella organization of Statutory Health Insurance (SHI) and the respective pharmaceutical company. Further details of the assessment process, including the statistical methods applied, can be found exemplarily online in journal articles [3, 4] and in the IQWiG method paper [5].

Non-small-cell lung cancer (NSCLC) is a therapeutic indication for which new drugs are frequently approved. Many NSCLC dossiers are thus submitted. In each dossier, the expected size of the target population is calculated via a complex multi-stage process by combining different patient characteristics (e.g. percentage of patients with NSCLC and with a specific tumour mutation). These characteristics are often selected on the basis of different assumptions (e.g. whether patients with or without prior treatment belong to the target population). In consequence, the total patient numbers may vary considerably between the different dossiers.

As the exact quantification of the target population can be important for price negotiations following the G-BA’s decision on added benefit, the present analysis aims to compare the different methodological steps applied to determine the target population in NSCLC dossiers.

## Methods

First, using the Summary of Product Characteristics (SPC), we checked the approved therapeutic indications and the corresponding patient characteristics reported in the NSCLC dossiers. We then identified and extracted information on common patient characteristics from the dossiers. For each characteristic, we then compared the methodological steps and the assumed percentages of affected patients across dossiers to identify those characteristics contributing the most to deviating target population numbers. We used information only if it was publicly accessible in the dossiers and did not fall under trade and business secrets.

Eligible dossiers were NSCLC dossiers assessed by IQWiG between 1 January 2011 (the start of the early benefit assessment process) and 31 December 2017 and subsequently published on the G-BA website.

## Results

Between 2011 and 2017, IQWiG assessed 24 NSCLC dossiers with 13 different new drugs: 8 protein kinase inhibitors and 5 monoclonal antibodies (Table 1).

**Table 1:** Dossiers assessed by IQWiG between 2011 and 2017 and published on the G-BA website

Drug	Year of publication	Drug class	Therapeutic indication according to SPC	Target population estimated by pharmaceutical company
Afatinib [19]	2013	Protein kinase inhibitor	*EGFR-TKI-naive* adult patients with *locally advanced and/or metastatic* NSCLC with activating *EGFR mutation(s)* [20]	1989 (1604 to 2374)
Afatinib (RE) [6]	2015	Protein kinase inhibitor		6539 to 16 450
Afatinib (ETI) [12]	2016	Protein kinase inhibitor	Adult patients with *locally advanced or metastatic* NSCLC of *squamous histology* progressing *on or after platinum-based chemotherapy* [20]	3793 to 4165
Alectinib [21]	2017	Protein kinase inhibitor	Adult patients with *ALK-positive advanced* NSCLC *previously treated with crizotinib* [22]	858 (264 to 1477)
Alectinib (ETI) [16]	2017	Protein kinase inhibitor	*First-line treatment* of adult patients with *ALK-positive advanced* NSCLC [22]	1599 (580 to 2463)
Atezolizumab [23]	2017	Monoclonal antibody	Adult patients *with locally advanced or metastatic* NSCLC after *prior chemotherapy* [24]	10,724 (8889 to 15,761)
Ceritinib [25]	2015	Protein kinase inhibitor	Adult patients with *ALK-positive advanced* NSCLC *previously treated with crizotinib* [26]	118 to 554
Ceritinib (RE) [8]	2016	Protein kinase inhibitor		95 to 568
Ceritinib (ETI) [15]	2017	Protein kinase inhibitor	First-line treatment of adult patients with *ALK-positive advanced* NSCLC [26]	430 to 850
Crizotinib [27]	2012	Protein kinase inhibitor	Adults with *previously treated ALK-positive advanced* NSCLC [28]	484
Crizotinib (RE) [7]	2016	Protein kinase inhibitor		76 to 427
Crizotinib (ETI 1) [10]	2015	Protein kinase inhibitor	*First-line treatment* of adults with *ALK-positive advanced* NSCLC [28]	678
Crizotinib (ETI 2) [11]	2016	Protein kinase inhibitor	Adults with *ROS1-positive advanced* NSCLC [28]	SP 1: 31 to 141^a^ SP 2: 11 to 56^b^
Dabrafenib/trametinib^c^ [17, 18]	2017	Protein kinase inhibitor	Adult patients with *advanced* NSCLC with a *BRAF V600 mutation* [29, 30]	SP 1: 128 to 259^a^ SP 2: 95 to 209^b^
Necitumumab [31]	2016	Monoclonal antibody	Adult patients *with locally advanced or metastatic EGFR expressing squamous* NSCLC who have *not received prior chemotherapy* for this condition [32]	6277 to 8707
Nintedanib [33]	2015	Protein kinase inhibitor	Adult patients with *locally advanced, metastatic or locally recurrent* NSCLC of *adenocarcinoma tumour histology after first-line chemotherapy* [34]	6592 to 15,148
Nivolumab [35]	2015	Monoclonal antibody	Adult patients with *locally advanced or metastatic squamous* NSCLC *after prior chemotherapy* [36]	4231 to 6015
Nivolumab (ETI) [13]	2016	Monoclonal antibody	Adult patients with *locally advanced or metastatic non-squamous* NSCLC *after prior chemotherapy* [36]	6567 to 9639
Osimertinib [37]	2016	Protein kinase inhibitor	Adult patients with *locally advanced or metastatic EGFR T790M mutation-positive* NSCLC [38]	SP 1: 1038 (562 to 1671)^b^ SP 2: 25 (10 to 51)^a^ SP 3: 16 (6 to 31)^b^
Osimertinib (RE) [9]	2017	Protein kinase inhibitor		1027 (529 to 2764)
Pembrolizumab [39]	2016	Monoclonal antibody	*Locally advanced or metastatic* NSCLC in adults whose *tumours express PD-L1 after prior chemotherapy* [40]	8795 to 14,679
Pembrolizumab (ETI) [14]	2017	Monoclonal antibody	*First-line treatment* of *metastatic* NSCLC in adults whose *tumours express PD-L1 *(*TPS ≥* 50%)* with no EGFR or ALK positive tumour mutations *[40]	4840 to 7982
Ramucirumab [41]	2016	Monoclonal antibody	Adult patients with *locally advanced or metastatic* NSCLC with disease progression *after platinum-based chemotherapy *[42]	11,008 (9888 to 12,282)

*ETI* extended therapeutic indication, *SP* subpopulation, *RE* reassessment after expiration of deadline

^a^Patients without prior (chemotherapy) treatment

^b^Patients with prior (chemotherapy) treatment

^c^Combination therapy

The number of NSCLC dossiers submitted increased considerably over time, from 2 in 2012–2013 to 17 in 2016–2017. For four drugs (afatinib [6], crizotinib [7], ceritinib [8], and osimertinib [9]), a second dossier was submitted after the initial deadline and the drug was reassessed, meaning that the G-BA’s initial appraisal decision was only valid for a certain period of time.

For six drugs, the extension of the therapeutic indication resulted in further dossier submissions and subsequent assessments (2 on crizotinib [10, 11], and one each on afatinib [12], nivolumab [13], pembrolizumab [14], ceritinib [15], and alectinib [16]).

A special case refers to two separate, but identical, dossiers and assessments for the same dual combination therapy (dabrafenib [17], trametinib [18]) submitted by the same company, which were treated as one dossier in the present analysis. In total, our data basis thus consisted of 23 dossiers.

Patient numbers and therapeutic indications differed considerably; only a few drugs were approved for the same indication (alectinib [22] and ceritinib [26], alectinib (ETI) [22] and crizotinib (ETI1) [28]). Accordingly, the information on the size of the specific target populations varied. The therapeutic indications reported in the SPCs covered a wide range of patient characteristics. While some of these characteristics applied to patients across all dossiers (e.g. diagnosis of lung cancer and NSCLC), others only applied to very specific patient populations in individual dossiers (e.g. patients with a BRAF-V600 mutation [29, 30]).

**Fig. 1:**
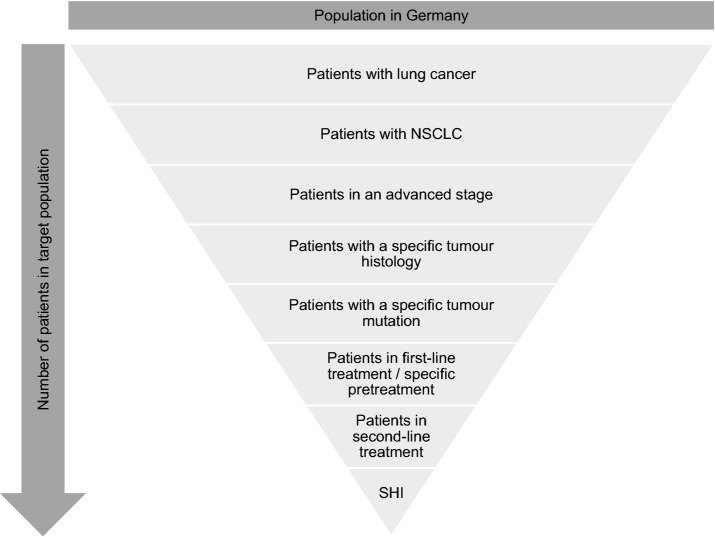
Most common patient characteristics considered by manufacturers when calculating the target populations in NSCLC dossiers. The order of the calculation steps can vary. SHI: patients insured in Statutory Health Insurance

Figure 1 shows the most common patient characteristics reported in the dossiers. The order of the characteristics considered in the calculation process varied. Moreover, not all characteristics were included in each dossier. However, all calculations of the target populations had in common that at the start of the calculation process, an initial population was determined on the basis of the information on the size of the general population in Germany as well as on the incidence and/or prevalence of lung cancer. The percentage of patients with specific characteristics in relation to the population of the previous step was subsequently calculated in a stepwise process (e.g. approximately 80% of all lung cancer patients suffer from NSCLC).

The patient characteristics, the methods applied for the determination of the respective patient populations, and the data sources used are explained below in more detail.

### Patients with lung cancer

The starting point for determining the number of patients with lung cancer was usually incidence and/or prevalence data or a combination of both. The data reported in the dossiers were either obtained from epidemiological registries (e.g. the German Centre for Cancer Registry Data at the Robert Koch Institute, RKI) or calculated on the basis of disease rates and the population size in Germany in a specific year.

As epidemiological data on patients with lung cancer were not available from RKI for the year of dossier submission, data from prior years were extrapolated for the dossiers. At RKI, incidence data were available for 2011 to 2017 and 5-year prevalence data were available for 2013 to 2016.

Moreover, the forecasts for the number of patients with lung cancer differed depending on the forecast for the general population numbers, the database used to determine the disease rate, and the statistical methods applied. In most of the dossiers regarded here, the extrapolation took place via linear regression [8, 12–15, 17, 18, 27, 35, 39] or fitted linear regression [16, 21, 23] assuming a constant incidence rate in men and a linear increase of the incidence in women. In four dossiers, no forecast was applied [10, 11, 31, 41].

Whether incidence, prevalence or their combination was chosen as an epidemiological measure in the dossiers differed depending on whether the target population included patients with prior treatment or treatment-naïve patients with advanced and/or metastatic disease (stage IIIB/IV). In some dossiers, more than one research question was considered, covering both patients with and without prior treatment. Where possible, the research questions are examined separately in the following text.

### Epidemiological data chosen if patients without prior treatment are in the focus

**Fig. 2:**
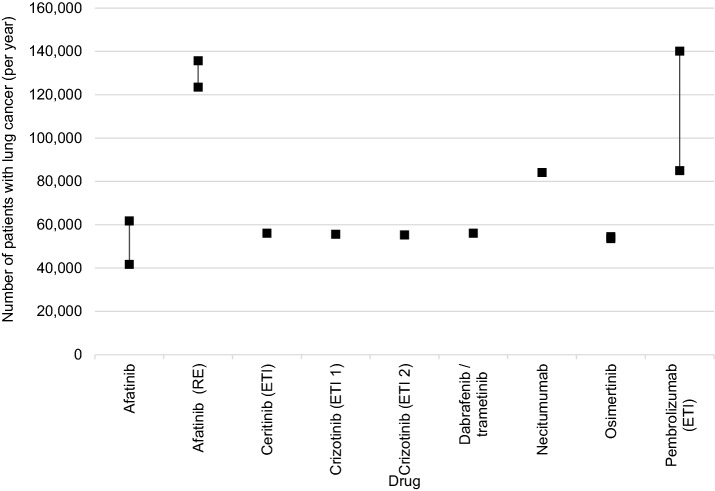
Epidemiological data chosen for patients with lung cancer if patients without prior treatment are in the focus. A range indicates the range of values presented in a dossier

The number of treatment-naïve patients with lung cancer was reported in nine dossiers [6, 10, 11, 14, 15, 17–19, 31, 37] and the epidemiological data chosen as the basis for calculation ranged between 41,723 and 140,136 patients with lung cancer per year (lower number: estimated 5-year prevalence 2013 [19]; higher number: estimated sum of 5-year prevalence 2016 and incidence 2017 [14]), see Fig. 2.

The most common epidemiological measure used in this context was the incidence of lung cancer (five dossiers) [10, 11, 15, 17, 18, 37]. The 5-year prevalence was used only in two dossiers [19, 31], as were combinations of incidence and prevalence measures [6, 14]. Different approaches were followed in this context: the 5-year prevalence of the previous year plus the incidence of the reference year [6] or the 5-year prevalence as the lower limit and the 5-year prevalence of the previous year plus the reference year’s incidence as the upper limit [14].

### Epidemiological data chosen if patients who received prior treatment are in the focus

**Fig. 3:**
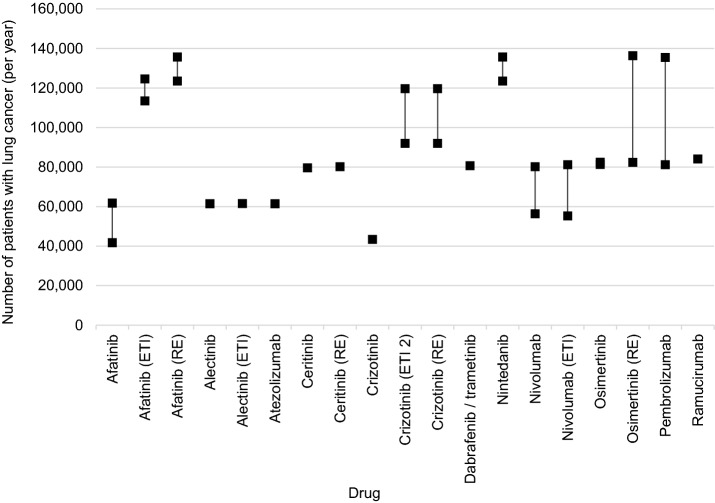
Epidemiological data chosen for patients with lung cancer if patients with prior treatment are in the focus. A range indicates the range of values presented in a dossier

The number of pretreated lung cancer patients was reported in 19 dossiers [6–9, 11–13, 17–19, 21, 23, 25, 27, 33, 35, 37, 39, 41] and the epidemiological data chosen as basis for the calculation ranged between 41,723 and 136,354 patients with lung cancer per year (estimated 5-year-prevalence 2017 and sum of 5-year-prevalence 2016 and incidence 2017) [19], see Fig. 3.

A common epidemiological measure used in this context was the 5-year prevalence (six dossiers, [8, 17–19, 25, 37, 41]. The incidence was used in four dossiers [16, 21, 23, 27]). In addition, patients diagnosed with lung cancer in previous years who had progressed to an advanced and/or metastatic disease stage (stage IIIB/IV) in the specific year of consideration were included in three dossiers [16, 21, 23]. A combination of both measures with different approaches was used in nine dossiers [6, 7, 9, 11–13, 33, 35, 39].

### Patients with NSCLC

**Fig. 4:**
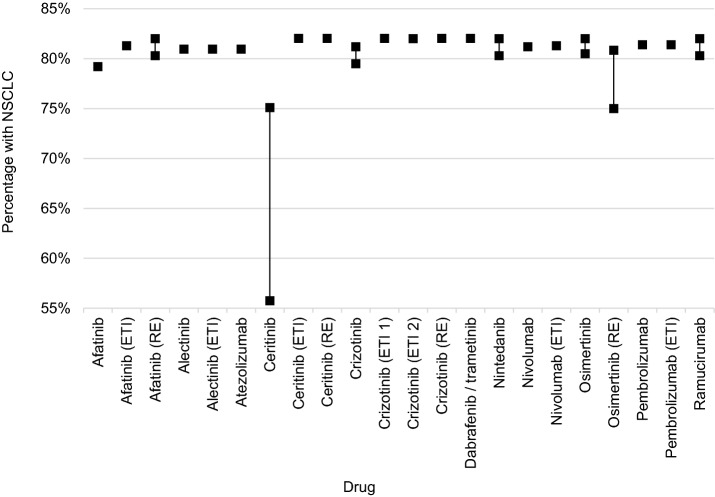
Percentage of NSCLC in all lung cancer patients (per year)

In each dossier, the percentage of patients with NSCLC in all lung cancer patients was derived from data of federal state cancer registries. This percentage ranged from 75 [9] to 82% (five dossiers [7, 8, 10, 15, 17, 18]) in the different dossiers (Fig. 4), with one outlier reporting a lower limit of 55.7% [25].

Different sources and observation periods were used in the dossiers to obtain the percentage of NSCLC in all patients with lung cancer. The main source for the majority of dossiers (20 dossiers) was the registry analysis by the German Tumour Centre’s Working Group in cooperation with the Network Quality Assurance through Clinical Cancer Registries (ADT/KoQK) as well as the Munich Cancer Registry (MCR) [6–19, 21, 23, 33, 35, 37, 39, 41]. The ADT/KoQK includes data on 210,076 newly diagnosed NSCLC and SCLC patients from 31 clinical registries in 11 German federal states for the years 2000 to 2014 [43]. The MCR includes data on 25,147 newly diagnosed NSCLC and SCLC patients collected by hospitals and physicians in Upper Bavaria and the town and county of Landshut [44, 45]. In the dossiers analysed here, data from the years 1998 to 2014 were used. Further sources included the commercial Tumour Registry Lung Cancer (four dossiers [6, 19, 33, 41]) and a varying number of federal state cancer registries (six dossiers [16, 21, 23, 25, 27, 31]). It should be noted, that because the dossiers related to different years, the periods analysed in the various registries also differed.

In eight dossiers [12–14, 19, 31, 35, 37, 39], mean values were calculated from different data sources, for example by averaging numbers of the ADT/KoQK registry analysis and the MCR; two dossiers used ranges [9, 37].

### Patients in a certain disease stage (locally advanced and/or metastatic NSCLC)

The therapeutic indication of the drugs assessed comprised adult patients with mostly advanced or locally advanced and/or metastatic NSCLC.

The pharmaceutical companies operationalized these populations as patients in stage IIIB or IV (IIIB/IV) according to the Union for International Cancer Control (UICC) disease stage classification [46].

Nintedanib is also approved for locally recurrent NSCLC [34].

The therapeutic indication for pembrolizumab from 2017 [40] only covers adult patients with metastatic NSCLC; in this case, the company operationalized patients as disease stage IV.

Twenty dossiers [6–13, 15–19, 21, 23, 25, 31, 35, 37, 39, 41] derived the percentage of patients in disease stage IIIB/IV from the sources mentioned below, which mainly include newly diagnosed patient populations with NSCLC. This was independent of whether the percentage derived applied to incident patients or prevalent ones.

A total of 17 of 20 dossiers referred to publications based on the ADT/KoQK registry [6–13, 15–19, 21, 23, 35, 37, 39] (percentage of approx. 60% with NSCLC and disease stage IIIB/V), eight dossiers [6, 9, 12, 13, 19, 35, 37, 39] referred to the publication of a cohort study [47] which reported a percentage of 50% in a newly diagnosed population of patients with NSCLC, five dossiers referred to the MCR (percentage of approx. 55%) [9, 13, 19, 35, 37], and 5 used other public tumour registries with percentages ranging between 57.6 and 78.5% [16, 21, 23, 25, 31]. In addition, three dossiers submitted analyses based on data from a commercial source and reported percentages of up to 90% in the respective patient population [6, 12, 19].

**Fig. 5:**
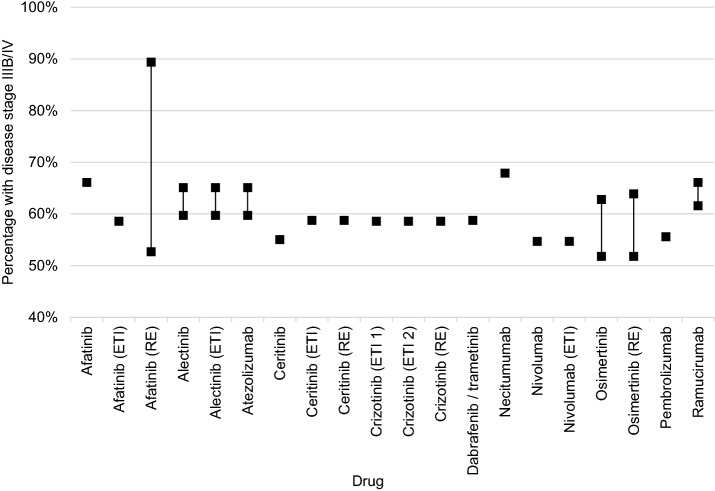
Percentage of disease stage IIIB/IV in all NSCLC patients. The mean or “most plausible” value is not presented if a range is presented

Ten of the 20 dossiers considered determined a mean value, as percentages were derived from different sources [9, 12, 13, 19, 25, 31, 35, 37, 39, 41], using different calculation methods; three dossiers used one source containing the most plausible percentage and established a range around this value with percentages from sources classified as less appropriate [16, 21, 23]. Across all dossiers, the percentages ranged between about 50 and 90% (Fig. 5).

### Patients with disease progression

Most companies did not separately consider the progression of a disease from an early stage to an advanced and/or metastatic stage (stage IIIB and/or IV). The number of patients with disease progression was often considered indirectly by taking the prevalence of the previous year as the initial population. However, the percentage of patients in disease stage IIIB and/or IV, which was applied to the initial population, was usually obtained from newly diagnosed patients; hence, this percentage only partly matched a prevalent patient population (see “Patients with lung cancer” section).

Only three dossiers [16, 21, 23] considered disease progression when calculating the target population. The percentage determined considered the number of patients in an early disease stage who later progressed to an advanced disease stage.

### Histology

According to the tumour classification system of the World Health Organization (WHO) [48, 49], the following histological types of NSCLC can be distinguished: adenocarcinoma, squamous-cell carcinoma, large-cell carcinoma, and NSCLC not otherwise specified. This classification is decisive for subsequent diagnostic and therapeutic action. Tumour histology was considered in six dossiers investigating therapeutic indications with histological specifications [6, 12, 13, 31, 33, 35]: two dossiers reported information on adenocarcinomas [6, 33], with percentages ranging between 61.2 and 66.0%; five dossiers reported ranges between 23.5 and 36.9% patients with squamous-cell carcinomas [6, 12, 13, 31, 35], and only one dossier reported percentages for large-cell carcinomas (5.5%) and NSCLC not otherwise specified (3.36%) [6]. The percentages presented in these six dossiers were based on market or health service research studies or on cancer registries.

It should be noted that a data analysis from 2016 based on the data of the ADT on lung cancer care in Germany showed a significant, unexplained increase in the percentage of adenocarcinomas between 2000 and 2014 [43].

### Patients with a tumour mutation

NSCLC can exhibit different tumour mutations, which influence the malignant phenotype of the tumour cells. Depending on the specific mutation, different targeted treatment options exist for the respective patient population. Such treatments were considered in 15 of the 23 NSCLC dossiers for the following mutations: anaplastic lymphoma kinase (ALK), rapidly accelerated fibrosarcoma-isoform B (BRAF), epidermal growth factor receptor (EGFR) and the C-ros oncogene 1 (ROS1). Percentages for different tumour mutations were mostly obtained from clinical, molecular biological, market research, or cohort studies.

The percentage of patients with ALK-positive NSCLC was reported in nine dossiers [7, 8, 10, 14–16, 21, 25, 27] and ranged between 1.6 and 13.5%, with nearly half of them [8, 16, 21, 25] reporting a range between 2.0 and 7.0%. Two approaches were used to determine the percentage: (1) determination of a percentage or a range that was weighted for the percentage of ALK mutations within the respective histological group, and (2) direct extraction of the percentage from the literature.

The percentage of patients with an EGFR mutation was reported in four dossiers [6, 9, 19, 37], with a wide range from 4.9 to 23.0% without consideration of any NSCLC histology. To determine this percentage, the dossiers also used the two approaches described above.

The percentage of patients with a BRAF mutation was reported in the dossiers on dual combination therapy (considered as one dossier) and ranged from 1 to 2% [17, 18]; the same range for ROS 1 mutations was reported in another dossier [11].

In two dossiers, the percentage reported was multiplied by the rate of patients tested for mutations in a routine care setting [7, 11], irrespective of the specific tumour mutation. By doing so, only those patients with a known mutation status were taken into account.

### Chemotherapy regimen/prior treatment

As outlined above, according to the SPC, most of the NSCLC drugs were approved for pretreated patients (18 dossiers [6–9, 11–13, 17–19, 21, 23, 25, 27, 33, 35, 37, 39, 41]); 11 of these dossiers provided further specifications [8, 9, 12, 13, 21, 23, 25, 33, 35, 39, 41], such as “after prior chemotherapy”, “after platinum-based chemotherapy”, “previously treated with crizotinib” or “prior treatment with EGFR-TKI”. A few drugs were approved for patients without prior treatment (five dossiers [10, 14, 15, 17, 31]).

The same stepwise process to examine the percentage of patients with prior treatment was used in eight dossiers [6, 12, 13, 19, 23, 35, 39, 41]: starting from the initial population (see above), the percentage of patients with systemic first-line treatment was determined. Then the percentage of patients with chemotherapy as first-line treatment and subsequently the percentage of patients with second-line treatment in the respective population of the previous step were determined. The same references are often cited in all three steps. The most frequently cited studies were a German prospective observational study [50, 51], the prospective observational EPICLIN lung study [52] and the LENS study [53], as well as evaluations by commercial sources.

**Fig. 6:**
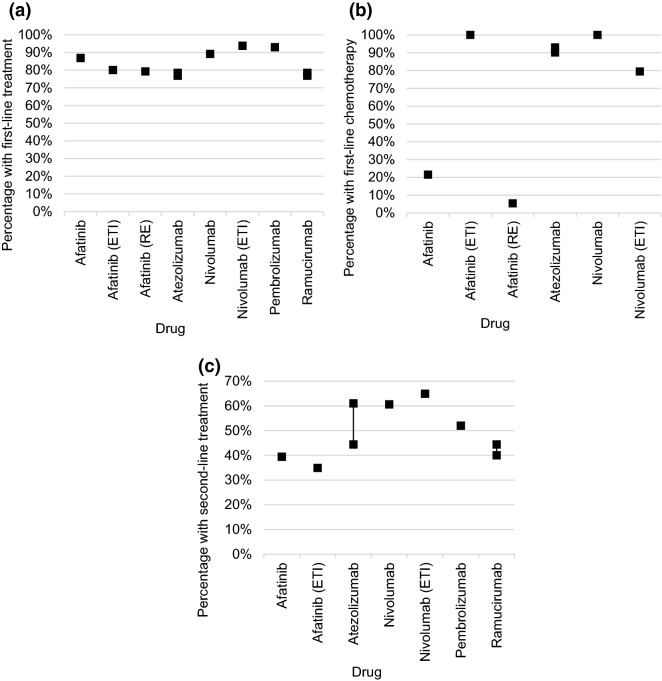
**a**–**c** Percentage of patients with first-line treatment (**a**), first-line chemotherapy (**b**) and second-line treatment (**c**)

The percentages reported for first-line treatment varied between 76.9 [23, 41] and 93.8% [13] (Fig. 6a).

Large variations were shown in the percentages reported for patients with chemotherapy as the first-line treatment (Fig. 6b), ranging from 5.4% based on an analysis by a commercial provider [6] to 100% based on the same source in a different dossier [12]. Various other numbers were reported, depending on the reference consulted: one dossier referring to the EPICLIN lung study reported a percentage of 90.1% [23]; a further dossier referring to the LENS study reported a percentage of 79.5% [13]; the same study was referred to by two other dossiers, but they reported a percentage of 100% [12, 35]. One dossier reported a percentage of 93% based on information from a commercial registry [23] and another reported a percentage of 21.5% and referred to a French study [19, 54].

The percentages reported for patients with second-line treatment ranged between 34.9 [12] (average of 22.0% reported in an analysis by a commercial provider and 47.7% reported in a German prospective observational study [50]) and 64.9% [13] reported in the LENS study (Fig. 6c).

## Discussion

Over 20 dossiers on new drugs for NSCLC have been submitted and assessed within the framework of early benefit assessments in Germany. During this time, the treatments became more and more targeted to specific patients groups. This development will probably continue in the next years, causing more complex calculations of the target populations with additional calculation steps. Because of this differentiation, the size of target populations varies considerably between dossiers and the target populations as a whole can only be compared to a limited extent. Comparability is only given for separate calculation steps.

### Patients with lung cancer

The variations in the incidence and 5-year prevalence data across dossiers can be partly explained by the fact that different reference years were used; results were thus affected by the increasing prevalence and incidence of lung cancer over time [55, 56]. The NSCLC dossiers analysed differed with regard to the epidemiological measures used to quantify the population of patients with lung cancer; the type of measure used also depended on whether the drug was approved for pretreated or treatment-naïve patients. The most commonly used measure for patients without prior treatment was lung cancer incidence, which causes uncertainty in two regards: first, the incidence does not include patients diagnosed in the previous year but not yet treated at the time of data collection. This could cause an underestimation of the target population; second, the incidence includes patients diagnosed late in the reference year but not treated until the following year. This could cause an overestimation. For this reason, combinations of incidence and (5 years) prevalence numbers or incidence measures were used in several dossiers. Regarding patients with prior treatment, a combination of the (5 years) prevalence of the previous year and the incidence of the reference year was the most common measure used. This approach follows the assumption that, in general, each patient diagnosed in the previous year(s) in addition to each patient newly diagnosed in the reference year is eligible for a (new) treatment. The sole use of the (5 years) prevalence of the reference year would not include all patients and cause an underestimation of the target population, as patients who died during the reference year are not considered, even though they could have received treatment in the same year before they died. Overall, especially the differences in the approaches chosen by the pharmaceutical companies for the calculation of the size of the initial population resulted in the wide range of numbers reported.

### Patients with NSCLC

With two exceptions [9, 25], the percentage of patients with NSCLC of all patients with lung cancer showed only little variation compared with other steps in the calculation procedure of the target population. One reason for variations might be caused by the apparent increase in this percentage over time [44]. Other reasons for varying NSCLC percentages could be the use of different data sources and different approaches to calculate the percentages. The data sources used were mainly registries, which may be affected by differences in completeness and by regional differences. Moreover, different patient classification criteria with regard to the histological type of cancer (NSCLC vs. SCLC) may also explain variations [57]. A more detailed description of the classification criteria used in early benefit assessments would be useful.

In the different dossiers, the percentage of patients with NSCLC was applied to the overall population of incident cases, prevalent cases or a combination of both measures. Registry analyses generally contain information on the time of diagnosis, i.e. on incidence, and hence lack information on prevalence (the reason for this is that the estimation of prevalence is impaired because deaths are often reported with a time lag). Due to different mortality rates between NSCLC and SCLC patients [44, 45], the transfer of the percentage of incident patients with NSCLC to prevalent data could yield different percentages.

### Patients with advanced and metastatic NSCLC

For this calculation step, the percentages reported in the dossiers ranged between 50 and 70%. Again, these differences can largely be explained by the use of different data sources and different methods to derive the percentage of patients with advanced and/or metastatic NSCLC. Data sources were predominantly (local) registries with varying levels of completeness, and also cohort studies and data from commercial sources.

Again, in several dossiers, the percentages were derived from a population of newly diagnosed patients and then transferred to the prevalent cases. However, it can be assumed that the percentages for (prevalent) patients with advanced/metastatic disease are considerably lower than for newly diagnosed patients due to the higher mortality rates.

### Patients with a specific histology

Dossiers reporting information on percentages of histological subgroups reported different percentages depending on the subgroup: The percentages reported for adenocarcinomas showed only minor variations (between 61.2 and 66.0%), but the percentages for squamous carcinoma ranged between 23.5 and 36.9%. Different histological classifications can partly explain these variations, as no standardized process for assigning morphological codes to histological groups exists [57, 58]. Again, differences could partly be explained by an increase of lung cancer over time [56]. As stated, a detailed description of classification criteria would be useful.

### Patients with a specific tumour mutation

Many different kinds of sources were used to determine the percentage of patients with a specific tumour mutation. The extent of variation between the percentages reported differed across the dossiers, also depending on the respective mutation.

There are various reasons that may explain the differences in the percentages of tumour mutations: first, different methods may be applied to identify mutations in study populations [47, 59] such as different screening tests (with varying sensitivity) or immunohistochemical staining methods or histological classifications. Methods of operationalization may also vary; for instance, some results referred to the number of patients, others to the number of cell samples. Second, it is uncertain whether study results from non-European countries [60–63] or results from small studies [60, 64] can be applied to the German health care context.

In addition, the percentages reported largely depend on tumour histology, especially for ALK and EGFR mutations. For instance, if a certain study with NSCLC patients included a higher percentage of patients with adenocarcinomas compared with the average NSCLC population in Germany, this could result in a considerably larger proportion of patients with tumour mutations. Different approaches used in the dossiers to determine the target population for ALK and EGFR mutations (mere extraction of available study data vs. generation of a percentage weighted for histological classifications) may explain further deviations.

### Patients in a specific line of treatment

The variations in this calculation step were due to the different populations that the percentages related to: for example, data from the same source (LENS study) were applied to patients with squamous carcinoma in one case and to patients with non-squamous carcinoma in the other. Moreover, the percentage of patients with second-line treatment was in one case related only to patients who received prior treatment, whereas in the other case it related to all patients included. The deviations in percentages of 5.4% and 100% for patients with first-line chemotherapy obtained from the same source of a commercial provider can only be partly explained by the different definitions of patient groups in the dossiers concerned [6, 12]: the percentage of 5.4% referred to patients receiving first-line chemotherapy in relation to all patients receiving first-line treatment, whereas the percentage of 100% referred to patients receiving chemotherapy as second-line treatment in relation to how many of these patients had received chemotherapy as first-line treatment.

In general, it is again debatable whether the percentages generated through these calculation steps can to the same extent be transferred to the number of incident and prevalent patients.

Overall, our findings show a wide range of therapeutic indications within NSCLC, which result in very specific patient groups. Hence, comparing absolute patient numbers is usually possible only to a limited extent. The calculation of patients in the target population is the result of a multi-stage process. Each calculation step contributes to an increasing variation of the final number of patients. The focus in the assessment of the dossiers is not only on the final size of the target population but also on each single calculation step. Therefore, in the assessment of dossiers, it is important to check on the basis of the SPC whether the calculation steps are complete and comprehensive. If important steps are missing or additional steps are implemented, their impact on the total size of the target population has to be analysed. For this analysis, a simple model is set up that combines the calculation steps and allows for a calculation with changing assumptions. After this, for each patient number or percentage of patients presented in a calculation step, the cited data sources are thoroughly assessed. The study population should match the target population, the timeframe of the data collection should not be outdated and the study results should be transferable to the German health care context as best as possible. In addition, the percentage of patients derived should be transferable to the patients in the previous calculation step. If different reliable data sources with deviant information are available, a range of minimum and maximum values will be assumed (and is often already presented in the dossier). This range will be used to test whether different assumptions cause deviations in the size or range of the final target population and to check the size of this impact. Finally, the values and assumptions chosen in the calculation steps are compared between dossiers within the same indication and reasons for deviations are analysed.

The process of the assessment and appraisal of the epidemiological information in a dossier by IQWiG and G-BA is further illustrated in a case study. Therefore, the dossier Ceritinib ETI [26] was chosen as in this indication only a few calculation steps are necessary to determine the target population. Ceritinib is indicated for first-line treatment of adult patients with ALK-positive advanced NSCLC [8]. According to that, the following calculation steps and percentages could be found in the dossier: as a starting point for further calculations, the incidence of lung cancer in the year 2011/2012 in Germany was chosen [55] and extrapolated to the year 2017 (56,095 patients). In the next step, the percentage of patients with NSCLC was assumed to be 82.03% based on an assessment of a (regional) German cancer registry [43]. Hereafter the stage IIIB/IV of the disease was assumed to be 58.76% according to the same cancer registry used in the previous step [43]. For the percentage of patients with ALK-positive mutation, another dossier in this indication was cited and the percentage was according to this document assumed to be 2.0%–3.9% [10, 11]. The percentage of patients receiving first-line treatment was assumed to be 92, 30% (based on a prospective non-interventional study in Germany [50, 51]) to 93.7% (based on the LENS-study [53]). After assuming a percentage of 86.07% patients insured in the statutory health insurance in Germany, the final size of the target population was calculated to include 430 to 850 patients.

In the assessment of this dossier, it has been concluded by IQWiG that the target population estimated by the pharmaceutical company does in total have a plausible size. Regarding the single calculation steps, it has been stated among others that the assumed percentage of patients with NSCLC and the percentage of patients in stage IIIB/IV of the disease are insecure because of limitations of the referred database. Moreover, it has been specified that according to scientific publications the assumed percentage of ALK-positive mutations could have a wider range.

Finally, the G-BA followed in his decisions to Ceritinib ETI the explanations and assumed percentages of the pharmaceutical company except for the percentage of patients with NSCLC and the percentage of patients in stage IIIB/IV. For both calculation steps, a wider range was assumed to take the insecurity more into account (percentage of patients with NSCLC: 79.34%–82.03%; percentage of patients in stage IIIB/IV: 51.8%–58.76%). The size of the target population was indicated with 366 to 850 patients in total [65].

Some of the factors causing variation between dossiers (e.g. increasing number of lung cancer and hence of NSCLC cases, especially of adenocarcinomas) can be attributed to the epidemiology of lung cancer and cannot be eliminated. Other factors can be attributed to the different calculation methods applied and data sources used; harmonizing these factors could contribute to a reduction in variations in the target populations reported for NSCLC. Especially the combination of percentages derived from different data sources leads to distortions in the calculation process if the percentages derived do not refer to the same populations of patients. Comprehensive and representative data sources allowing for an adequately specific representation of newly diagnosed patients with NSCLC, as well as their treatments and treatment outcomes over time, are so far lacking in Germany. Since 2013, all federal states have been obliged to set up clinical cancer registries (§65c SGB V) but this has not yet been fully implemented. Consequently, as long as no representative clinical cancer registries or large and sophisticated health services research studies are available, the combination of percentages from different data sources is a viable way to obtain information on the target population in a specific therapeutic indication. However, it is important that the dossiers provide detailed information on their data sources and methodological approaches.

In summary, the calculation methods applied and the target population sizes reported in NSCLC dossiers vary considerably. A homogenisation with regard to the databases and calculation methods used to determine the target population in NSCLC would be helpful to reduce variations.
